# Lymphoma-Like T Cell Infiltration in Liver Is Associated with Increased Copy Number of Dominant Negative Form of TGFβ Receptor II

**DOI:** 10.1371/journal.pone.0049413

**Published:** 2012-11-07

**Authors:** Weici Zhang, Masanobu Tsuda, Guo-Xiang Yang, Koichi Tsuneyama, Xiao-Song He, Aftab A. Ansari, William M. Ridgway, Ross L. Coppel, Zhe-Xiong Lian, Patrick S.C. Leung, M. Eric Gershwin

**Affiliations:** 1 Division of Rheumatology, Allergy and Clinical Immunology, University of California Davis, Davis, California, United States of America; 2 Diagnostic Pathology, Graduate School of Medicine and Pharmaceutical Science, University of Toyama, Toyama, Japan; 3 Department of Pathology, Emory University School of Medicine, Atlanta, Georgia, United States of America; 4 Division of Immunology, Allergy and Rheumatology, University of Cincinnati College of Medicine, Cincinnati, Ohio, United States of America; 5 Department of Microbiology, Monash University, Melbourne, Victoria, Australia; 6 Institute of Immunology, Hefei National Laboratory for Physical Sciences at Microscale and School of Life Sciences, University of Science and Technology of China, Hefei, China; Johannes Gutenberg University of Mainz, Germany

## Abstract

Hepatosplenic T cell lymphoma (HSTCL) is a distinct and lethal subtype of peripheral T cell lymphoma with an aggressive course and poor outcome despite multiagent chemotherapy. Contradictory literature, an unknown etiology, and poor response to treatment highlight the need to define the malignant process and identify molecular targets with potential for successful therapeutic interventions. Herein, we report that mice homozygously expressing a dominant negative TGFβRII (dnTGFβRII) under the control of the CD4 promoter spontaneously develop lymphoma-like T cell infiltration involving both spleen and liver. Splenomegaly, hepatomegaly and liver dysfunction were observed in homozygous dnTGFβRII mice between 10 weeks and 10 months of age associated with a predominant infiltration of CD4^−^CD8^−^TCRβ^+^NK1.1^+^ or CD8^+^TCRβ^+^NK1.1^−^ T cell subsets. Notch 1 and c-Myc expression at the mRNA levels were significantly increased and positively correlated with the cell number of lymphoid infiltrates in the liver of dnTGFβRII homozygous compared to hemizygous mice. Further, 2**×**10^4^ isolated lymphoma-like cells transplant disease by adoptive cell transfers. Collectively, our data demonstrate that increased copy number of dnTGFβRII is critical for development of lymphoma-like T cell infiltration.

## Introduction

Transforming growth factor beta (TGFβ) is a multifunctional protein that acts as an important regulator of cell growth, proliferation, differentiation, morphogenesis and inflammation. TGFβ exerts biological effects by ligation of its cognate cell surface TGFβ receptors with activation of downstream effectors including the TGFβR Smad family [1], [2], [3]. Alterations of specific components involved along the TGFβ signaling pathway results in loss of TGFβ receptor function and disruption of the intracellular TGFβ signaling cascade. Such loss of TGFβR function is implicated in the pathogenesis of aortic pathology, various cancers and fibrotic and inflammatory disease [1], [2], [4]. There is a reduction of TGFβRII expression in Burkitt’s lymphoma [5] and advanced cutaneous T cell lymphomas [6], [7]. Similarly, loss of surface TGFβRII or mutations in the TGFβRII gene have been reported in human T cell malignancies [8] and colorectal cancer [2], [9], [10], suggesting that abnormal expression of TGFβRII is associated with malignant progression. Thus, abnormalities in the TGFβ signaling pathway may be involved in the molecular pathogenesis of lymphoid malignancy. In the study herein, we generated homozygous dominant negative TGFβRII mice in which a 2-fold increase in expression of dnTGFβRII transgene was detected under control of the CD4 promoter. We demonstrated that mice homozygous for dnTGFβRII spontaneously developed lymphoma-like T cell infiltration involving both the spleen and liver with a significantly elevated pro-oncogene expression of Notch 1 and c-Myc. Further, we demonstrated that 2**×**10^4^ lymphoma-like cells were able to transplant disease by adoptive cell transfer.

## Materials and Methods

### Animals

Homozygous dnTGFβRII IL-6^−/−^ mice and dnTGFβRII mice were generated by cross breeding hemizygous dnTGFβRII and homozygous IL-6^−/−^ mouse strains as described [11]. Two breeding methods for colonies were applied in this study. 1) To maintain the dnTGFβRII transgene, male hemizygous dnTGFβRII, hemizygous dnTGFβRII IL-6^−/−^ and hemizygous dnTGFβRII p40^−/−^ mice were backcrossed onto female C57BL/6 (B6), IL-6^−/−^ and p40^−/−^ mice, respectively. The dnTGFβRII transgene is easier to maintain in hemizygosity because of severe inflammatory bowel disease when homozygous. 2) To generate homozygous mice, hemizygous dnTGFβRII, dnTGFβRII IL-6^−/−^ and dnTGFβRII p40^−/−^ mice were intercrossed. For example, hemizygous male dnTGFβRII IL-6^−/−^ mice were bred with hemizygous female dnTGFβRII IL-6^−/−^ to obtain homozygous dnTGFβRII IL-6^−/−^ mice. The resulting offspring mice were individually screened for p40, IL-6 and TGFβRII dominant negative genotype by PCR using prepared genomic DNA as previously described [11], [12]. Rag1 deficient mice of a C57B6 background (Ly5.2) were bred onto the congenic C57BL/6-Ly5.1-Pep3b (B6 Ly5.1) (The Jackson Laboratory, Bar Harbor, ME) mice to obtain Ly5.1 Rag1-deficient mice. All mice were maintained in individually ventilated cages under specific pathogen-free conditions and fed sterile rodent Helicobacter Medicated Dosing System (three-drug combination) diets (Bio-Serv, Frenchtown, NJ). Experiments were performed following approval from the University of California Animal Care and Use Committee.

### Flow Cytometry for Phenotyping and Intracellular Cytokine Analysis

Mononuclear cells were isolated from the spleen and liver tissue using density gradient centrifugation utilizing Histopaque-1.077 (Sigma-Aldrich, St. Louis, MO). Anti-mouse CD16/32 (clone 93, Biolegend) was used to block the Fc receptor prior to staining. The mononuclear cells were stained with fluorochrome-conjugated antibodies including APC-eFluor® 780–conjugated anti-TCR-β (clone H57–597, eBiosciences), Alexa Fluor 647-conjugated anti-CD19 (clone eBio1 D3, eBiosciences), PerCP-conjugated anti-CD4 (clone RM4–5, Biolegend), FITC-conjugated anti-CD8a (clone 53-6.7, Biolegend) and PE-conjugated anti-NK1.1 (clone PK136, BD-PharMingen, San Diego, CA).

For intracellular cytokine analysis, mononuclear cells isolated from splenic tissues were cultured in media containing Leukocyte Activation Cocktail, with BD GolgiPlug™ (BD Pharmingen, San Diego, CA) for 4 hours. Cells were stained for cell surface markers with PerCP anti-CD8a (clone 53-6.7, Biolegend), APC-conjugated anti-TCR-β (clone H57–597, eBiosciences), and APC-eFluor® 780-conjugated anti-NK1.1 (clone PK136, eBiosciences). After staining, the cells were fixed with BD Cytofix/Cytoperm™ solution and permeabilized with BD Perm/Wash™ buffer (BD Pharmingen, San Diego, CA). Aliquots of these cells were stained with FITC- or PE- conjugated antibodies against IFN-γ, IL-2 as well as their respective isotype control antibodies. Stained cells were analyzed using a FACScan flow cytometer (BD Bioscience) that was upgraded by Cytec Development (Fremont, CA), which allows for five-color analysis. Data were analyzed utilizing CELLQUEST software (BD Bioscience). Appropriate known positive and negative controls were used throughout.

### Histopathology

Immediately after sacrifice, the lung, spleen, liver and colon were harvested, fixed in 4% paraformaldehyde (PFA) at room temperature for 2 days, embedded in paraffin, and cut into 4- micrometer sections. The liver sections were de-paraffinized, stained with hematoxylin and eosin (H&E), and evaluated using light microscopy.

### Real time RT-PCR Analysis

To determine the relative mRNA levels of the genes including Notch-1, the Notch-1 ligands DLL1/DLL4, the Hes-1 gene which involved in Notch signaling, and for purposes of control the gene PTEN that is associated with DNA repair were quantitated in RNA extracted from liver tissues of the appropriate strains of mice. Total RNA was extracted from individual liver tissues using the QIAGEN RNeasy Mini Kit (Qiagen, Valencia, CA). For real time PCR analysis, 1 µg of total RNA was reverse transcribed and then quantified on an ABI ViiA™ 7 Real-Time PCR System (Applied Biosystems, Foster City, CA). Amplification was performed for 40 cycles in a total volume of 20 µl and products were detected using SYBR Green (Applied Biosystems, Foster City, CA). The relative level of expression of each target gene was determined by normalizing its mRNA level to an internal control gene GAPDH.

### Clonality Analysis

The clonal T cell expansions were identified by CDR3-length analysis of TCRVβ gene segments as described [13].

### Relative Quantification of Transgene Copy Number

Relative quantification of transgene copy number was established as previously described [14]. Briefly, genomic DNA was obtained from mouse ear using the QIAamp DNA Mini Kit (QIAGEN, Valencia, CA), and then diluted to 2 ng/ml. Amplification was performed for 40 cycles in a total volume of 10 µl and products were detected using SYBR Green (Applied Biosystems, Foster City, CA) and quantified on an ABI ViiA™ 7 Real-Time PCR System. The primer sequences for dominant negative TGFβRII were as follows. Forward: GCTGCACATCGTCCTGTG, Reverse: ACTTGACTGCACCGTTGTTG. A single copy gene within the mouse genome, Survival Motor Neuron (SMN) gene [15], [16] was used as a reference gene. The primer sequences for SMN were as follows. Forward: TGGGAGTCCATCCATCCTAA, Reverse: CGACTGGGTAGACTGCCTTC. Relative quantification methods (2^−ΔΔCt^ methods) were used for relative quantification of transgene in mouse genome by normalizing target gene to the reference gene SMN.

### Adoptive Cell Transfer

For adoptive cell transfer, mononuclear cells were collected from the liver tissues of dnTGFβRII mice by density gradient centrifugation using Histopaque-1.077. Eight-week- to ten-week-old recipient Ly5.1Rag1^−/−^ mice were injected intravenously with 2×10^4^ or 2×10^5^ hepatic mononuclear cells from the donor mice. For adoptive CD8 T cell transfer, mononuclear cells were collected from the liver tissues of hemizygous dnTGFβRII mice without lymphomatous lesions, stained with antibodies including FITC-conjugated anti-CD8b (clone H35-17.2, eBioscience), PE-conjugated anti-NK1.1 (clone PK136, BD-PharMingen, San Diego, CA), PE-Cy5-conjugated anti-CD8a (clone 53-6.7, eBiosciences) and Alexa Fluor 750–conjugated anti-TCR-β (clone H57–597, eBiosciences). Cells were sorted using a 10-parameter MoFlo cell sorter (Cytomation, Fort Collins, CO). The purity of sorted CD8ab T cells was >96%. Aliquots of 1×10^6^ sorted CD8ab T cells were intravenously injected into individual 8–10-week-old Ly5.1Rag1^−/−^ mice.

### Statistical Analysis

The data are presented as the mean ± SEM. Two-sample comparisons were analyzed using the two-tailed unpaired t-test. The correlation between two parameters was analyzed using Spearman Correlation Method. A value of p<0.05 was considered statistically significant.

## Results

### Lymphoma-like T Cell Infiltration in Intercrossed dnTGFβRII IL-6^−/−^ Littermates

**Figure 1:**
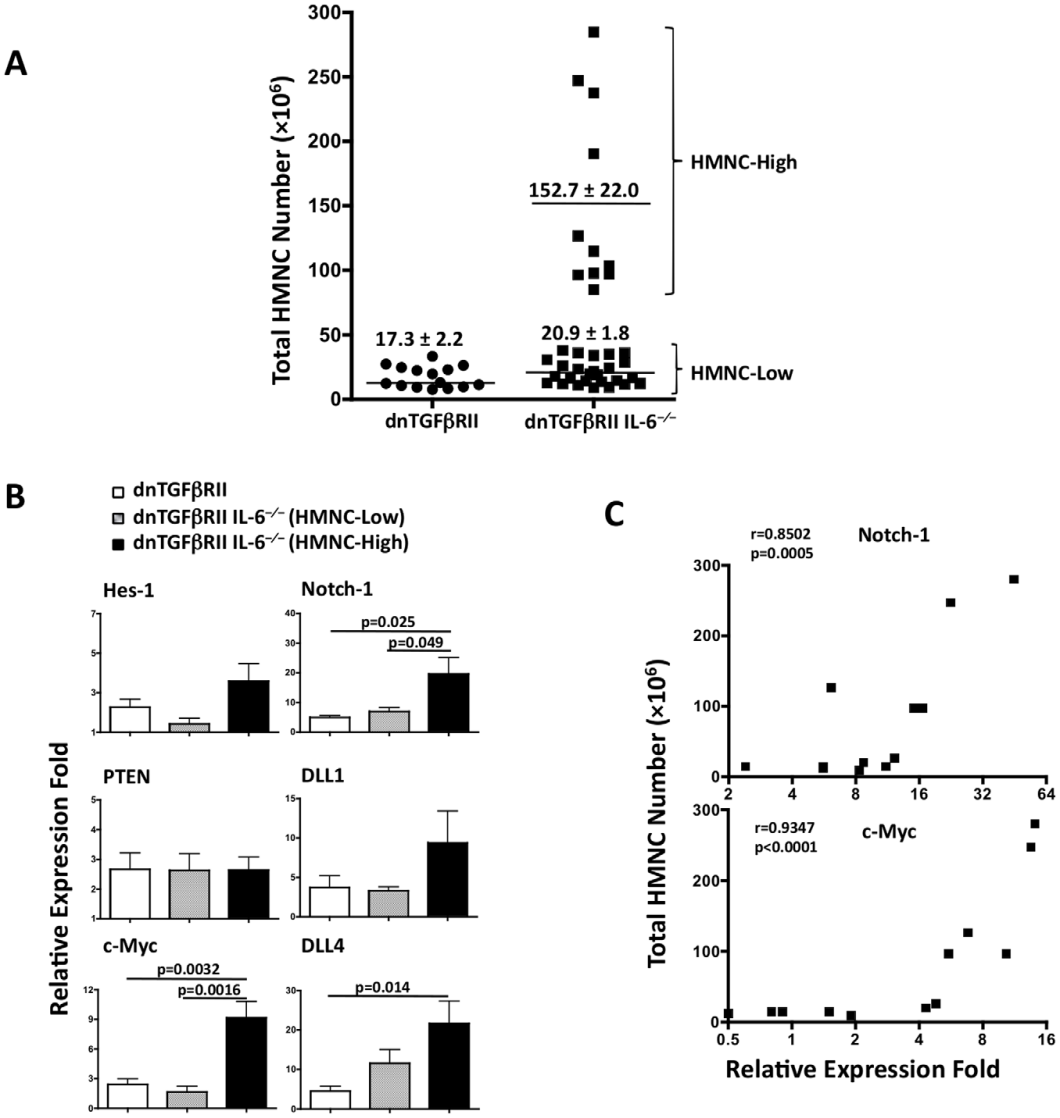
Lymphoma-like T cell infiltration developed in interbred dnTGFβRII IL-6^−/−^ mice. A. Number of liver infiltrating HMNCs in interbred dnTGFβRII IL-6^−/−^ mice in comparison with hemizygous dnTGFβRII mice. Data presented as Mean ± SEM; dnTGFβRII, n = 15; dnTGFβRII IL-6^−/−^, n = 37. B. The expression of Notch-1 and c-myc mRNA levels are significantly higher in livers of dnTGFβRII IL-6^−/−^ mice with higher HMNC counts (HMNC-high) than dnTGFβRII IL-6^−/−^ mice with lower HMNC counts (HMNC-low). Data presented as Mean ± SEM, n = 6. C. Spearsman correlation between HMNC counts and pro-oncogene expression.

Our lab has previously documented that deletion of the IL-6 gene from the hemizygous dnTGFβRII mice significantly improved colitis as indicated by substantially reduced intestinal lymphocytic infiltration, reduced diarrhea and increased body weight, while maintaining the autoimmune cholangitis. In the follow-up study of cholangitis in this mouse model, we expanded this colony by intercrossing hemizygous TGFβRII IL-6^−/−^ litters. In this process hemizygous dnTGFβRII^+/−^ IL-6^−/−^ mice were generated along with homozygous TGFβRII^+/+^ IL-6^−/−^ and TGFβRII^−/−^IL-6^−/−^ littermates, in a ratio that follows Mendel’s law of segregation. When individual animals generated by intercrossing were examined for the liver infiltrating mononuclear cells (MNCs), two distinct subsets of littermates with TGFβRII transgene were found to have dramatically different (6- to 12-fold) numbers of liver infiltrating MNC (Figure 1A). One of the subsets, approximately one third of the TGFβRII littermates, had 152.7±22.0×10^6^ hepatic mononuclear cells (HMNCs), while the rest of the animals only had 20.9±1.8×10^6^ HMNCs. Such massive HMNC increase suggested a lymphoma-like disease. Therefore we examined the mRNA levels of the lymphoma-related proto-oncogenes involved in the Notch-1 signal pathway, including Notch-1, DLL1/4, Hes-1, PTEN and c-Myc in the liver tissues of individual animals. Among these genes, the relative mRNA levels of Notch-1 and c-Myc were the most pronounced and significantly increased in the littermates with massive HMNCs when compared to those with fewer HMNC infiltration (c-Myc: 9.2±1.7 vs. 1.7±0.6, n = 6, p = 0.0016) (Figure 1B), or compared with the hemizygous dnTGFβRII mice (c-Myc: 9.2±1.7 vs. 2.4±0.6, n = 6, p = 0.0032) (Figure 1B). A significant positive correlation was detected between the number of HMNCs and Notch-1 (r = 0.8502, p = 0.0005, Spearsman correlation), and between HMNCs and c-Myc in interbred mice (r = 0.9347, p<0.0001, Figure 1C). These results indicate that lymphoma-like T cell infiltration occurred in the interbred dnTGFβRII^+^ IL-6^−/−^ mice. Since the lymphoma-like HMNCs are seen in approximately one third of the dnTGFβRII^+^ IL-6^−/−^ mice generated by intercross breeding that resulted in a mixture of littermates with hemizygous (+/−) and homozygous (+/+) dnTGFβRII gene, but not seen in pure hemizygous dnTGFβRII^+/−^ IL-6^−/−^ litters generated without intercrossing, the results also suggest that the lymphoma-like T cell infiltration occurred in mice with the homozygous dnTGFβRII gene.

### Characterization of the Lymphoma-like HMNCs

**Figure 2:**
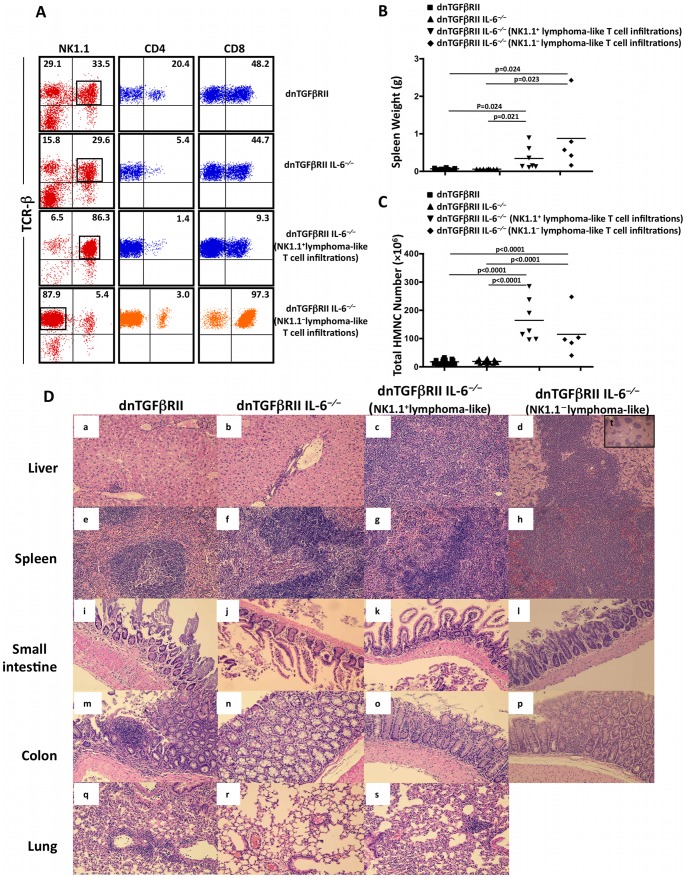
Histological features and immunophenotypes of lymphoma-like T cell infiltration. A. Flow cytometric analysis of HMNCs from dnTGFβRII and dnTGFβRII IL-6^−/−^ mice with and without lymphatomous lesion. The numbers above the plots indicate the frequency of TCRβ^+^NK1.1^−^ and TCRβ^+^NK1.1^+^ cells (left panels), the frequency of CD4 positive cells (middle panels) and the frequency of CD8 positive cells (right panels). Cells shown in the middle and right panels were gated on TCRβ^+^NK1.1^−^ or TCRβ^+^NK1.1^+^ populations as indicated in the left panels. B. The spleen weight of dnTGFβRII, dnTGFβRII IL-6^−/−^ and dnTGFβRII IL-6^−/−^ mice with a predominant NK1.1 positive or negative phenotype at age of 24–40 weeks. C. The total HMNC counts of dnTGFβRII, dnTGFβRII IL-6^−/−^ and dnTGFβRII IL-6^−/−^ mice with a predominant NK1.1 positive or negative phenotype at age of 24–40 weeks. D. Representative H&E stained sections of tissue sample including liver (a–d), spleen (e–h), small intestine (i–l), colon (m–p) and lung (q–s) were prepared from dnTGFβRII and dnTGFβRII IL-6^−/−^ mice at age of 24–40 weeks (a–s,**×**200; t,**×**40). Typical diffuse lymphomatous lesions were found in liver (c) and spleen (g) of dnTGFβRII IL-6^−/−^ mice with a predominant NK1.1^+^ phenotype, while large focal lymphomatous lesions were found in liver (d,**×**200)(t,**×**40) and spleen (h) of dnTGFβRII IL-6^−/−^ mice with a predominant TCRβ^+^ NK1.1^−^ phenotype. No obvious lymphomatous lesions were found in lung, small intestine and colon in dnTGFβRII and dnTGFβRII IL-6^−/−^ mice.

**Figure 3:**
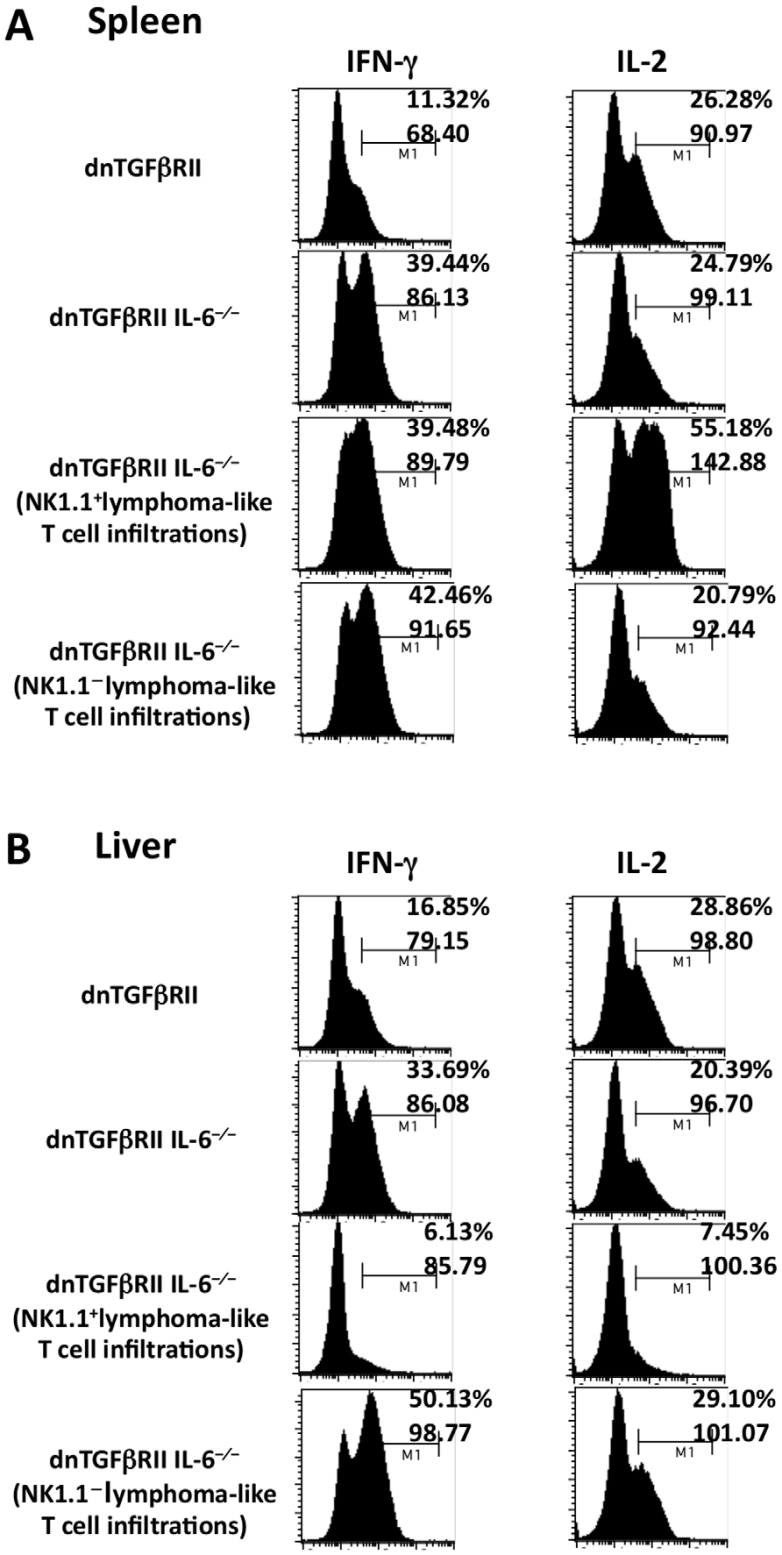
Cytokine profile of dnTGFβRII IL-6^−/−^ mice with lymphomatous lesions. Intracellular cytokine production in hepatic (A) and splenic (B) T cells was determined by flow cytometry. The percentages and MFI of cytokine-producing T cells are shown. The frequency of IFN-γ and IL-2-producing T cells is lower in liver of dnTGFβRII IL-6^−/−^ mice with a predominant NK1.1^+^ phenotype than dnTGFβRII IL-6^−/−^ mice with a predominant NK1.1^−^ phenotype (IFN-γ^+^ T: 6.13% vs. 50.13%; IL-2^+^ T: 7.45% vs. 29.10%). The data are representative of three independent experiments.

**Figure 4:**
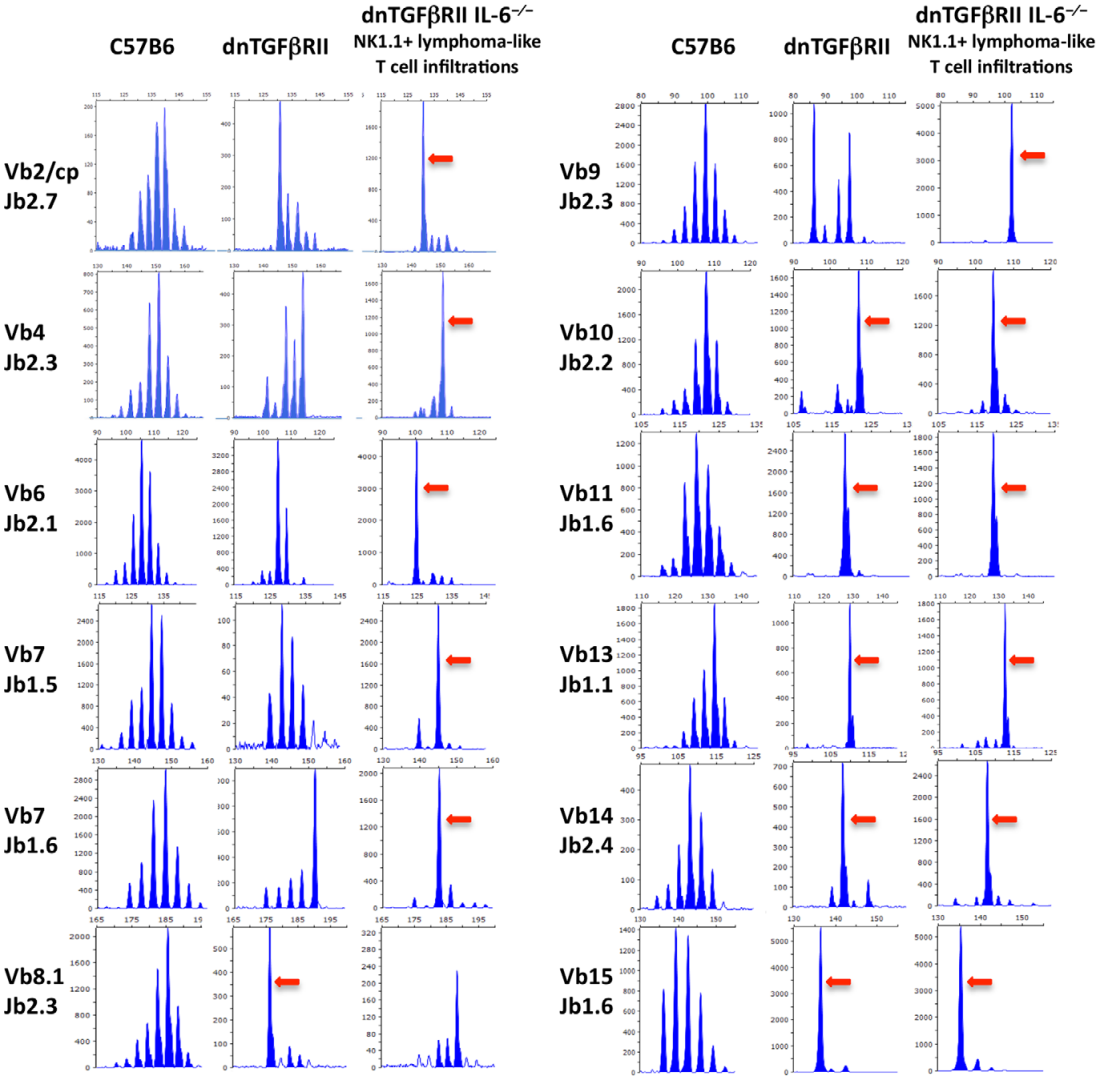
Comparison of CDR3 region of the TCR β family between dnTGFβRII and dnTGFβRII IL-6^−/−^ mice. The arrows indicate clonal expansion of specific Vβ. C57B6 mice were used as negative control. With this technique, if there is no detectable T cell expansion within a Vβ spectrum, a Gaussian distribution of CDR3 lengths is observed. In contrast, clonal expansions are observed as a perturbation of this Gaussian distribution.

Flow cytometric analysis was performed to determine the phenotype of the lymphoma-like HMNCs. As shown in Fig. 2A, in mice with greatly elevated HMNC infiltration (HMNC high), or hepatic lymphoma, the HMNCs were comprised of two major phenotypes. Seven out of 12 lymphoma-like mice were characterized by the predominance of CD4^−^CD8^−^TCRβ^+^NK1.1^+^ cells (termed NK1.1), whereas the other 5 lymphoma-like mice had a predominant CD8^+^TCRβ^+^NK1.1^−^ T cell subset (termed NK1.1^−^) in the liver tissues. The percentage of HMNCs with these phenotypes ranged from 70–98%, indicating that these mice developed lymphoma-like T cell infiltration. Mice with HMNCs in these two phenotypes had a significant increase in splenic weight (Figure 2B) and hepatic MNC count (Figure 2C) compared to mice without such predominant HMNC phenotypes. Histological examinations were performed on lymphoid (Figure 2D, a-h) and non-lymphoid (Figure 2D, i-s) organs. Massive atypical lymphoid infiltration was observed in the grossly enlarged spleen and liver, but not in the small intestine or colon, of the mice with these predominant HMNC phenotypes (Figure 2D). Marked lymphoid aggregation was only found in the lung of 1/7 mouse with predominant CD4^+^TCRβ^+^NK1.1^+^ infiltrates. In liver sections, a diffuse infiltration with atypical lymphocytes was observed in mice with CD4^−^CD8^−^TCRβ^+^NK1.1^+^ HMNC phenotype, whereas the CD8^+^TCRβ^+^NK1.1^−^ phenotype was associated with focal lymphoid aggregates (Figure 2D, d:×200, t:×40). These histological changes were not present in the inbred dnTGFβRII IL-6^−/−^ littermates without massive HMNC infiltration. NK1.1^+^ lymphoma-like T cells isolated from the liver had a markedly reduced ability to produce IFN-γ and IL-2 compared to NK1.1^−^ lymphoma-like cells and HMNCs of non-lymphoma dnTGFβRII IL-6^−/−^ mice (Figure 3A), while such difference was not seen in the spleen (Figure 3B). We next determined the clonality of the T cells in HMNC by examining T cell receptor Vβ repertoire (Vβ1–20) CDR3 length and joining beta (Jβ1.1–1.6 and Jβ 2.1–2.7). The results demonstrated that both lymphoma-like and non-lymphoma dnTGFβRII IL-6^−/−^ mice displayed restricted Vβ repertoires. However, Vβ2/cp Jβ2.7, Vβ4 Jβ 2.3, Vβ7 Jβ1.5 and Vβ9 Jβ2.3 were highly restricted in lymphoma-like mice compared to the non-lymphoma littermates, indicating that expanded lymphoid populations are clonally heterogeneous (Figure 4).

### The Development of Lymphoma-like T Cell Infiltration was Associated with Homozygosity of dnTGFβRII Gene

**Figure 5:**
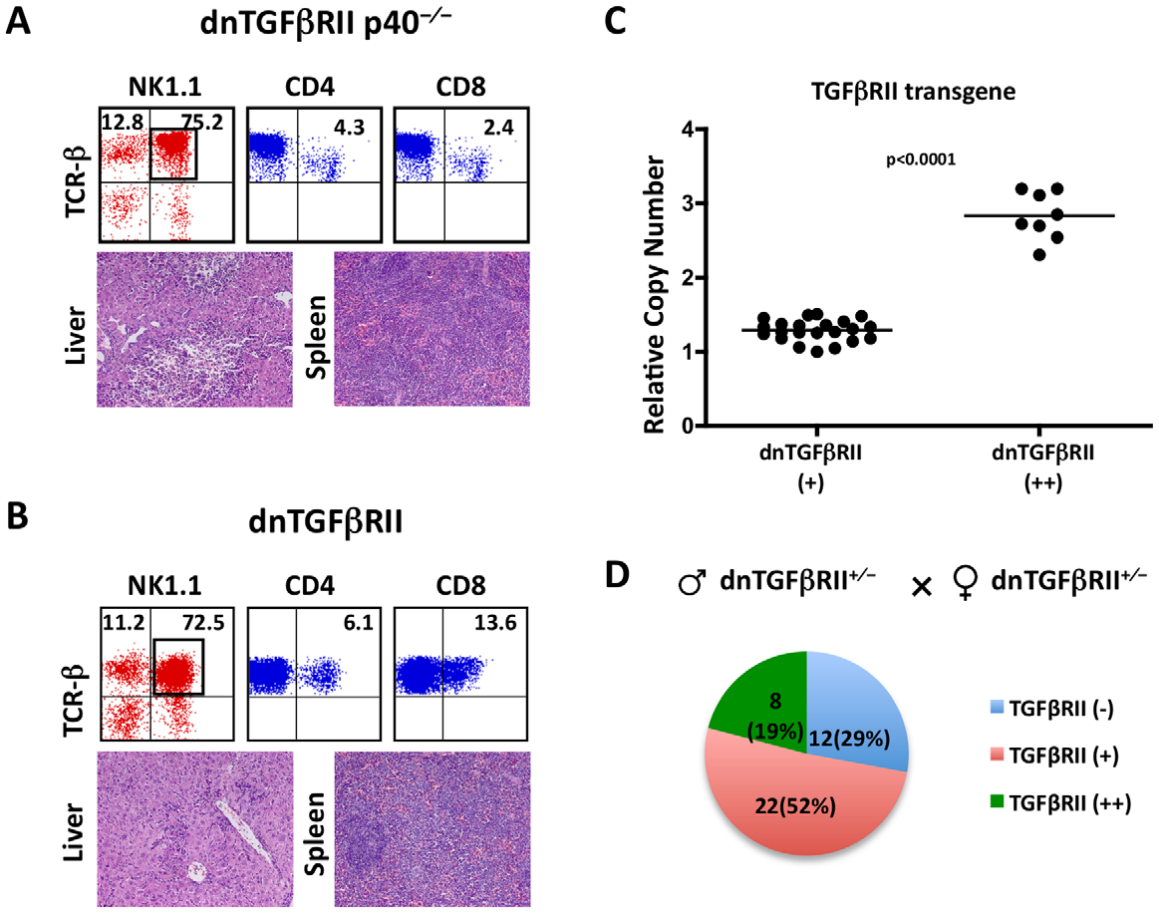
Lymphoma-like T cell infiltrations were only found in homozygous dnTGFβRII p40^−/−^ and dnTGFβRII mice. A. Representative immunophenotype of hepatic lymphocytes (upper panels) and H&E stained sections (lower panels) from inbred dnTGFβRII p40^−/−^ mice with lymphomatous lesions at age of 10 weeks. CD4 and CD8 double negative TCRβ^+^NK1.1^+^ cells are predominant in the liver of dnTGFβRII p40^−/−^. Typical diffuse lymphomatous lesions were found in liver and spleen. B. Representative immunophenotype of liver infiltrating lymphocytes (upper panels) and H&E stained sections from inbred dnTGFβRII mice with lymphomatous lesions at age of 12 weeks. C. Relative copy number of dnTGFβRII transgene detected by real-time PCR. D. The percentage of homozygous and hemizygous offspring from hemizygous TGFβRII parents.

It is critical to exclude the possibility that the observed severe HMNC infiltration in dnTGFβRII IL-6^−/−^ littermates actually reflects severe autoimmune liver inflammation rather than hepatic lymphoma. We have previously demonstrated that the autoimmune cholangitis in dnTGFβRII mice is mediated by Th1 cells, and that abrogation of the Th1 pathway by depleting IL-12 p40 gene completely protect dnTGFβRII mice from both cholangitis and colitis [12]. In order to further confirm the relationship between dnTGFβRII homozygosity and lymphoma-like T cell infiltration and to differentiate lymphoma from severe autoimmune cholangitis, we intercrossed our previously reported hemizygous dnTGFβRII p40^−/−^ mice, which were cholangitis-free, to generate a mixture of hemizygous and homozygous dnTGFβRII p40^−/−^ littermates with a ratio that follows Mendel’s law of segregation. As expected, substantially increased HMNCs (9.4±0.12×10^7^) were observed in the liver tissues of two out of seven, similar to the expected one-third frequency, intercrossed dnTGFβRII p40^−/−^ mice at the age of 10 weeks. Liver pathology examination demonstrated massive atypical lymphoid hepatic infiltration in these mice, indicating that introduction of a homozygous dnTGFβRII gene into the cholangitis-free dnTGFβRII p40^−/−^mice resulted in development of lymphoma-like T cell infiltration (Figure 5A).

Finally we generated intercrossed dnTGFβRII mice to determine if homozygous dnTGFβRII gene alone is sufficient to cause lymphoma-like T cell infiltration. Severe IBD in female hemizygous dnTGFβRII gene mice reduced the rate of fertility. Therefore only 3 interbred littermates were obtained. In one out of these 3 littermates a predominant CD4^−^CD8^−^TCRβ^+^NK1.1^+^ cell subset was found in the liver, which also demonstrated massive atypical lymphoid hepatic infiltration at 12 weeks (Figure 5B). Taken together, the findings in the intercrossed dnTGFβRII IL-6^−/−^, dnTGFβRII p40^−/−^ and dnTGFβRII mice indicate that dnTGFβRII homozygosity is associated with occurrence of lymphoma-like T cell infiltration.

### Increased Copy Number of dnTGFβRII is Associated with the Development of Lymphoma-like T Cell Infiltration

To directly confirm that lymphoma-like disease occurs with a higher copy number of dnTGFβRII gene in homozygous dnTGFβRII mice, we determined the relative dnTGFβRII transgene copy number in intercrossed dnTGFβRII littermates by quantitative real-time PCR. Given the fact that the parental generations are hemizygous, Mendel’s law of segregation predicts that the entire litter would be comprised of 50% of hemizygous (dnTGFβRII^+/−^), 25% homozygous (dnTGFβRII^ +/+^) and 25 percent negative (dnTGFβRII^−/−^) in the dnTGFβRII transgene. We determined the relative levels of the transgene in genomic DNA in comparison to a reference single copy gene SMN in 42 mice derived from 7 litters generated by intercrossing mice that carried hemizygous dnTGFβRII gene. These included 5 litters of intercrossed dnTGFβRII IL-6^−/−^ mice, 1 litter of dnTGFβRII p40^−/−^ mice and 1 litter of dnTGFβRII mice. In 22 mice (52%), the relative transgene level was 1.29±0.03; in 8 mice (19%) the relative transgene levels was 2.83±0.11, while in 12 mice (29%) the transgene was not detected (Figure 5C). Among the 8 mice with the higher level of dnTGFβRII transgene, 4 mice died around 12 weeks, two mice had splenomegaly, hepatomegaly and jaundice with an 18-fold increase of total HMNCs at age 12 weeks compared to the lower transgene level controls; the last two mice had an approximate 3-fold higher number of HMNCs at age of 15 weeks. Importantly, all mice with lymphomatous lesions, including dnTGFβRII and dnTGFβRII p40^−/−^ mice shown in Figure 5A and 5B, had higher levels of TGFβRII transgene than those without lymphomatous lesions (2.41±0.22, n = 8 vs. 1.27±0.03, n = 15, p<0.0001). There was a highly significant positive correlation between hepatic cellular infiltrates and the copy number of dnTGFβRII transgene (r = 0.9342, p<0.0001), indicating that abrogation of TGFβ signaling by higher copy number of dnTGFβRII contributes to the emergence of T cell lymphoma-like T cell infiltration.

### Adoptive Cell Transfer to Rag1^−/−^ Mice

**Figure 6:**
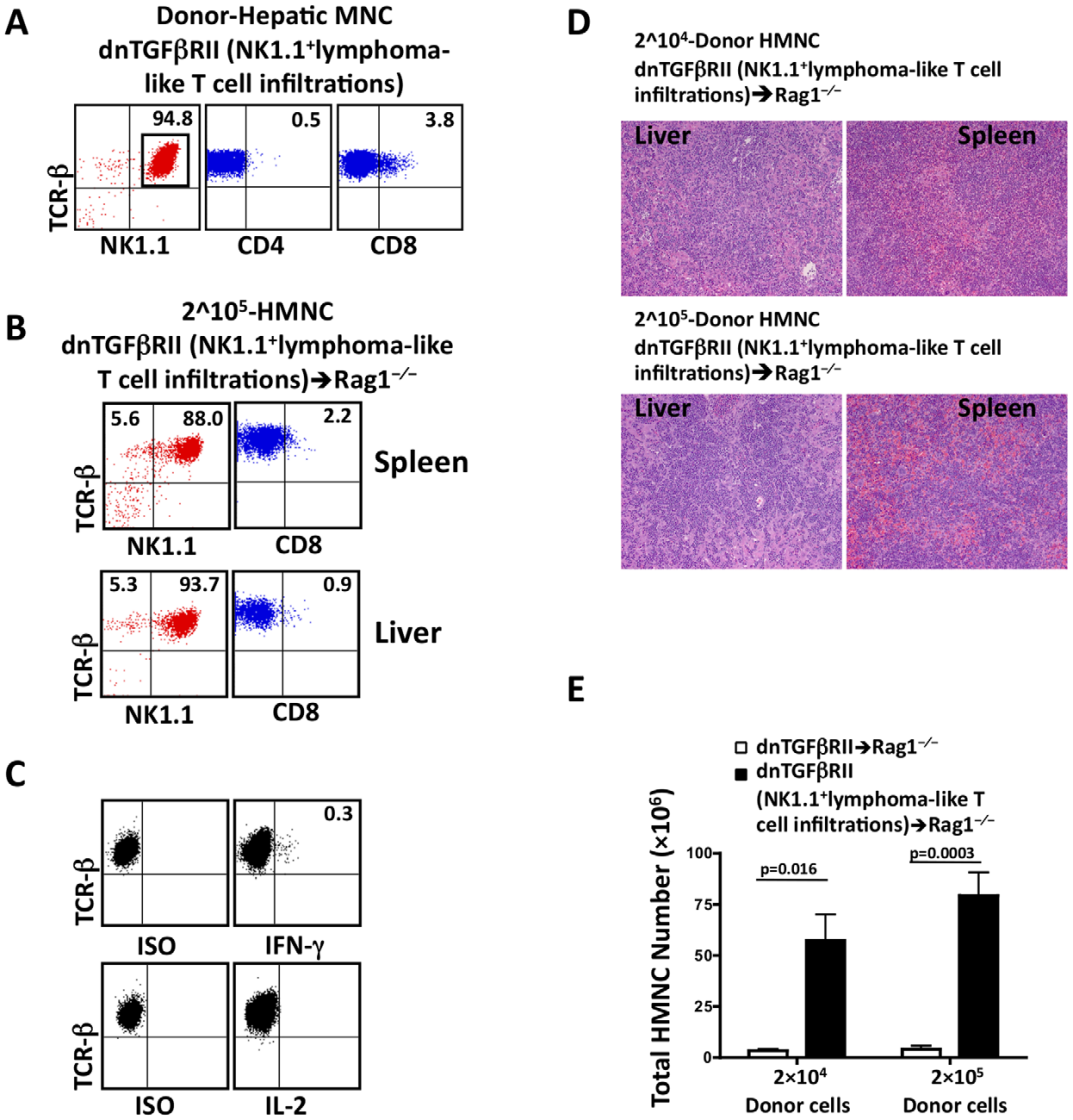
Lymphoma-like T cell infiltration is transplantable into Rag1^−/−^ mice. A. Flow cytometric analysis of HMNCs from donor mouse showing a TCRβ^+^NK1.1^+^CD4^−^CD8^−^ phenotype. B. Representative flow cytometric analysis of splenic and hepatic MNCs from recipient mice 6 weeks post-transfer. C, Intracellular IFN-γ and IL-2 production. D. H&E stained spleen and liver sections from Rag1^−/−^ recipient mice 6 weeks post-transfer of 2×10^4^ or 2×10^5^ HMNCs from inbred dnTGFβRII mice with lymphomatous lesions. E. Total HMNCs in Ly5.1Rag1^−/−^ recipient mice six weeks post-transfer. Ly5.1Rag1^−/−^ mice were adoptively transferred with 2×10^4^ or 2×10^5^ hepatic mononuclear cells from inbred dnTGFβRII mice with (n = 4) or without (n = 3) lymphomatous lesions, respectively.

To evaluate whether the lymphoma-like T cell infiltration in dnTGFβRII mice are transferable, we carried out standard adoptive transfer studies. We reported previously that transferring CD8^+^ T cells from hemizygous dnTGFβRII mice induced changes consistent with autoimmune cholangitis in Rag1^−/−^ mice only when the number of transferred cells reached one million [17]. We thus isolated HMNCs from hemizygous and homozygous dnTGFβRII mice and transferred 5- to 50-fold fewer cells (2×10^4^ or 2×10^5^) than the previous transfer studies [18], [19] into Rag1^−/−^ mice. The frequency of T cells expressing NK1.1 phenotype was approximately 95% in the liver of homozygous TGFβRII donors, 95% of TCRβ^+^NK1.1^+^ cells were CD4 and CD8 double negative (Figure 6A). Six weeks after intravenous injection, pathological and phenotypic changes identical to that seen in the donor homozygous mice were observed in spleen and liver in 8/8 recipient mice (Figure 6B and 6D). Histopathological studies revealed massive atypical lymphoid cell infiltrates and hepatocellular damage in recipient mice, even in mice that received as few as 2×10^4^ HMNCs from TGFβRII homozygous mice (Figure 6D). Consistent with the loss of cytokine function noted in the donor lymphoma-like cells, T cells from the recipient mice showed reduced cytokine production (Figure 6C). In contrast, no obvious lymphoid cell infiltrates were found in Rag1^−/−^ recipients of donor cells from hemizygous TGFβRII mouse (Figure 6D and 6E). The recipients of homozygous TGFβRII donor cells had significantly more HMNCs than those receiving the hemizygous donor cells (2×10^4^ cell group: 57.3±12.7×10^6^, n = 4 vs. 3.3±0.8×10^6^, n = 3; *p* = 0.016); (2×10^5^ cell group: 79.3±11.3×10^6^, n = 4 vs. 4.1±1.5×10^6^, n = 3; *p* = 0.0025). These results indicate that the lymphoma-like T cell infiltration in the homozygous TGFβRII mice can be efficiently transferred.

### Adoptive Transfer of Hepatic CD8ab Cells from TGFβRII Hemizygous Mice Resulted in Lymphoma-like Infiltration

**Figure 7:**
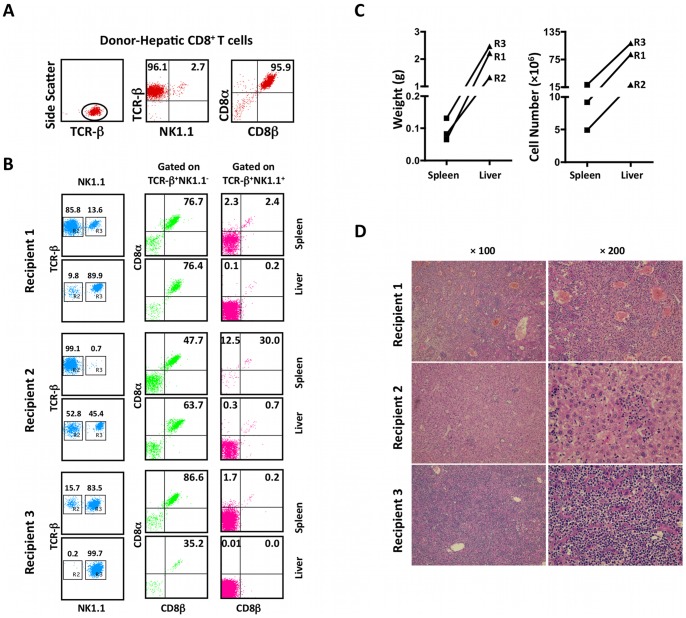
CD4 and CD8 double negative T cells were detected in Ly5.1Rag1^−/−^ mice six weeks after adoptively transferred with one million hepatic CD8ab T cells from hemizygous dnTGFβRII mice (lymphomatous lesion-free). A. Flow cytometry analysis demonstrated the purity of hepatic CD8αβ T cells from hemizygous dnTGFβRII mice. The numbers in the plots indicate the percentage of cells. B. Flow cytometric analysis of splenic and hepatic mononuclear cells of recipient mice at 6 weeks post-transfer. Three Ly5.1Rag1^−/−^ mice were adoptively transferred with 1×10^6^ hepatic CD8αβ T cells from hemizygous dnTGFβRII mice. TCRβ staining was gated on CD45.2^+^ cells. The numbers in the plots indicate the percentage of cells. C. Weight and total MNC counts of spleen and liver from Ly5.1Rag1^−/−^ recipients 6 weeks post-transfer. D. H&E staining sections of liver tissues from Ly5.1Rag1^−/−^ recipients six weeks post-transfer. R1, Recipient 1; R2, Recipient 2; R3, Recipient 3;

Since two distinct phenotypic T cell expansions were found in the TGFβRII homozygous mice, we addressed whether they were derived from the same precursors and transited through a TCRβ^+^NK1.1^−^ stage before they became NK1.1^+^ T cells during the terminal stage [20]. Flow cytometry sorted populations of hepatic CD8αβ cells from TGFβRII hemizygous mice were adoptively transferred to Rag1^−/−^mice (10^6^ sorted CD8 T cells per mouse, n = 3). The purity of the sorted CD8αβ cells was >95% as assessed by flow cytometric analysis (Figure 7A). Recipients were euthanized six weeks after adoptive transfer. Lung, heart, kidney, intestine, colon, spleen and liver were removed and examined histologically. The wet weight of spleen and liver were determined as shown in Figure 7C. Two of the three recipients had increased liver mass and cell count of mononuclear cells in both spleen and liver. Only 53% of MNCs from recipient 2 retained the TCRβ^+^NK1.1^−^ phenotype, while 45% of donor cells became CD4 CD8 double negative TCRβ^+^NK1.1^+^ in liver (Figure 7B). The frequency of TCRβ^+^NK1.1^+^ was higher in liver compared to that in spleen (Figure 7B). Histology revealed that bridging necrosis with severe to massive atypical lymphoid infiltration and hepatocellular damage were found in recipient 1 and recipient 3. On the other hand, only mild focal necrosis with mild lymphoid infiltration was observed in recipient 2. Our data collectively suggest that CD8 T cells from dnTGFβRII mice possess a capability to develop an NK1.1^+^ phenotype (Figure 7) with high pathogenic potential. In contrast, no NK1.1 positive T cells turned into NK1.1 negative T cells in immunodeficient Rag1^−/−^ mice (Figure 6).

## Discussion

The TGFβ signaling pathway plays an important role in T cell development and proliferation, naïve T cell homeostasis, peripheral T cell tolerance and effector T cell differentiation [20], [21]. To date, different strategies have been employed to generate mice with a T cell-targeted disruption of TGFβ signaling. In these models, consequences of TGFβ defects are limited to T cells. Mice that have a CD4-Cre-mediated deletion of a TGFβRII allele [20] develop a progressive lymphoid infiltration into multiple organs before 5 weeks of age [22]. Inserting a truncated TGFβRII under a CD2 promoter/enhancer in mice results in a CD8 T cell lymphoproliferative disorder with small lymphocyte infiltration [23]. Expression of a dominant-negative form of TGFβRII (dnTGFβRII) under a CD4 promoter without a CD8 silencer leads to spontaneous activation and differentiation of both CD4 and CD8 T cells and development of autoimmune diseases at 4–6 months of age [24]; the different pathological outcomes demonstrated that the TGFβ signaling pathway is not completely abrogated by the expression of the dominant negative form of TGFβRII.

We previously reported that hemizygous dnTGFβRII mice manifest autoimmune cholangitis with elevated Th1 cytokines in serum and liver [25]. In the present study, we unexpectedly found lymphoma-like disease in mice homozygous for dnTGFβRII. Several lines of evidence support of the linkage of this disease with lymphoma: First, in mice homozygous for dnTGFβRII 70–98% of hepatic MNCs were CD4^−^CD8^−^TCRβ^+^NK1.1^+^ or CD8^+^TCRβ^+^NK1.1^+^ cells. Second, a significantly increased expression of the proto-oncogenes Notch-1 and c-Myc, which correlates significantly with the number of HMNCs in mice homozygous for dnTGFβRII, compared to hemizygous dnTGFβRII mice. This observation has to be taken with caution partly because Myc and Notch can be induced upon normal T cell activation [26], [27]. Third, histological analysis demonstrated massive atypical lymphoid cell infiltration in the grossly enlarged spleen and liver from the homozygous dnTGFβRII, but not hemizygous dnTGFβRII mice. Fourth, adoptive transfer of very small numbers of infiltrating cells (2×10^4^) isolated from the liver of homozygous dnTGFβRII, but not from hemizygous dnTGFβRII mice, resulted in massive atypical lymphoid cell infiltration with CD4^−^CD8^−^TCRβ^+^NK1.1^+^ phenotype in Rag1^−/−^ recipients, which were identical to that of the donor. However, our present data here do not clearly discriminate lymphoma from inflammatory T-cell infiltration. NKT cells are most abundant in the liver. TGFβ signaling is critical for the differentiation of NKT subsets [28], but how TGFβ signaling involved in the regulation and differentiation of NKT in our mice model with abrogated TGFβRII needs to be further elucidated.

Oligoclonal expansion of T cells was detected in the liver of homozygous and hemizygous dnTGFβRII mice by clonality analysis, indicating that the expanded T cells are heterogeneous. Of note, clonality is not equivalent to malignancy, since benign and inflammatory conditions may show monoclonal rearrangement [29], [30]. Our finding of heterogeneous clonal expansion of lymphoma-like T cell in homozygous dnTGFβRII mice is consistent with previously documented heterogeneous clonal restrictions of T cell populations in patients with angioimmunoblastic T-cell [31] and cutaneous T-cell lymphoma [32]. Moreover, our clonality results support a previous study in AKR/J mice demonstrating that restricted TCR Vβ repertoire and lack of CDR3 conservation displayed thymic lymphomas [33]. In addition, lymphoma-like changes develop in dnTGFβRII mice with a skewed but polyclonal TCR repertoire, which is in agreement with a recently published study in which TCR-diversity suppressed development of mature T-cell lymphoma [34]. The clonal competition hypothesis might be a possible explanation for outgrowth of atypical lymphocytes in such a skewed TCR repertoire situation. Another possible explanation for local atypical infiltrates in our experimental system could be outgrowth of activated T cells accumulated in liver and mutations in certain oncogenes such as p53 [35]. The liver is a “graveyard” that actively sequesters activated and eventually apoptotic T cells [36]. However, activated T cells without the regulation of TGFβ signaling might undergo cell divisions rather than apoptosis due to mutations in oncogenes, resulting in outgrowth of atypical massive T cells. Further studies will be required to characterize the outgrowth of atypical lymphocytes in detail, including selections of malignant clones, deep sequencing and potential gene mutations to elucidate the underlying mechanism.

Patients with various autoimmune diseases have demonstrated an increased risk of developing non-Hodgkin lymphoma and multiple myeloma. Previous studies have also shown that loss of response to TGFβ is associated with the progression of different types of malignancies including T-cell lymphomas [7], [8]. Since it has been previously implicated that the expression of a single copy of dnTGFβRII transgene does not completely block TGFβ signaling in T cells, we reasoned that a second copy of dnTGFβRII transgene in homozygous mice further suppresses the downstream signaling, resulting in the development of T cell lymphoma. The pathological and immunological presentation in these lymphoma mice resembles the main clinical features in patients with HSTCL, although to date no γδ^+^ T-cell lymphomas have been found in homozygous dnTGFβRII mice. Strikingly, although elevated circulating IL-6 was reported to correlate with adverse clinical features and survival in non-Hodgkin lymphoma [37], [38], while clinical trials have showed anticancer effects of IL-12 on cutaneous T cell lymphoma [39], genetic depletion of IL-6 or IL-12p40 did not rescue outgrowth of lymphoma-like T cell in homozygous dnTGFβRII mice indicating that dnTGFβRII homozygosity is critical for the outgrowth of lymphoma-like T cells.

Peripheral T-cell lymphomas (PTCL) are rare and aggressive malignancies that are distinct from the more common cutaneous T-cell lymphomas. Hepatosplenic T cell lymphoma (HSTCL) is a distinct and lethal subtype of peripheral T-cell lymphoma with an aggressive clinical course and a dismal outcome despite multiagent chemotherapy. HSTCL likely arises from cytotoxic T-cells that express the γδ T-cell receptor type. However, it is important to note that an αβ T-cell phenotype has been described increasingly in HSTCL [40], [41], [42], [43]. Lymphoma cells usually have the following phenotype: CD2^+^, CD3^+^, CD4^−^, CD5^−^, CD7^+^ and CD8^−^. The World Health Organization has updated the classification of lymphomas, which has led to the application of a more stringent criteria for the diagnosis of enteropathy-associated T-cell lymphoma (EATL) [44]. However, the challenges in understanding and treating PTCLs remain, since published literature consists mostly of case reports. A wide range of pathologic subdivisions with varied clinical features also impedes systematic study on PTCL. Our findings highlight a potential role for TGFβ signaling in the development of HSTCL.

Consistent with the previous work by Marie and colleagues [20], we found that in the hemizygous TGFβRII mice an expanded subset of T cells expressed the NK1.1 marker, although lymphoma was not found in these mice. However, when we adoptively transfer the dysfunctionally activated TGFβRII-CD8 NK1.1^−^ T cells from these mice into the immunocompromised micro-environment in the Rag1^−/−^ recipients without regulatory T and B cells, expression of NK1.1 and disease pathology were observed (Figure 7), suggesting that expression of NK1.1 marker is associated with enhanced pathogenic potential. Although some lymphomas are phenotypically and genotypically of T cell origin, there are also lymphomas that are positive for the CD56 marker and are of NK cell origin [45]. NK cell markers are frequently expressed in HSTCL and other types of T cell lymphomas except for nasal and extranodal NK/T-cell lymphomas [46]. In comparison to CD56^−^ T-cell lymphoma, the CD56^+^ NK-like T cell lymphomas demonstrated an aggressive clinical course [45], [47] associated with a poor prognosis [48]. Our present study suggests that the acquisition of the NK1.1 cell surface marker by dnTGFβRII-CD8 T cells resulted in a highly pathogenic population that leads to development of T cell lymphoma with an aggressive clinical course. We speculate that blockage of the transition from NK1.1^−^ to NK1.1^+^ T cells could be a potential strategy for the management of lymphoma disease.

In summary, our data demonstrate that several features of human HSTCL are manifested in homozygous dnTGFβRII mice, suggesting that selective CD4 targeted functional abrogation of TGFβRII by increased copy number of dominant negative form of TGFβRII in mice can serve as a model of HSTCL for studying the disease mechanism and therapeutic strategies.
